# Semi-Automatic Segmentation of Optic Radiations and LGN, and Their Relationship to EEG Alpha Waves

**DOI:** 10.1371/journal.pone.0156436

**Published:** 2016-07-06

**Authors:** Emmanuelle Renauld, Maxime Descoteaux, Michaël Bernier, Eleftherios Garyfallidis, Kevin Whittingstall

**Affiliations:** 1 Department of Nuclear Medecine and Radiobiology, Faculty of Medicine and Health Science, University of Sherbrooke, Sherbrooke, Qc, Canada; 2 Department of Diagnostic Radiology, Faculty of Medicine and Health Science, University of Sherbrooke, Sherbrooke, Qc, Canada; 3 Sherbrooke Connectivity Imaging Lab (SCIL), Computer Science Department, Faculty of Science, University of Sherbrooke, Sherbrooke, Qc, Canada; 4 Centre Hospitalier Universitaire de Sherbrooke (CHUS), Sherbrooke, Qc, Canada; 5 Centre d’Imagerie Moléculaire de Sherbrooke (CIMS), Centre de Recherche du CHUS, Sherbrooke, Qc, Canada; University of Montreal, CANADA

## Abstract

At rest, healthy human brain activity is characterized by large electroencephalography (EEG) fluctuations in the 8-13 Hz range, commonly referred to as the alpha band. Although it is well known that EEG alpha activity varies across individuals, few studies have investigated how this may be related to underlying morphological variations in brain structure. Specifically, it is generally believed that the lateral geniculate nucleus (LGN) and its efferent fibres (optic radiation, OR) play a key role in alpha activity, yet it is unclear whether their shape or size variations contribute to its inter-subject variability. Given the widespread use of EEG alpha in basic and clinical research, addressing this is important, though difficult given the problems associated with reliably segmenting the LGN and OR. For this, we employed a multi-modal approach and combined diffusion magnetic resonance imaging (dMRI), functional magnetic resonance imaging (fMRI) and EEG in 20 healthy subjects to measure structure and function, respectively. For the former, we developed a new, semi-automated approach for segmenting the OR and LGN, from which we extracted several structural metrics such as volume, position and diffusivity. Although these measures corresponded well with known morphology based on previous post-mortem studies, we nonetheless found that their inter-subject variability was not significantly correlated to alpha power or peak frequency (p >0.05). Our results therefore suggest that alpha variability may be mediated by an alternative structural source and our proposed methodology may in general help in better understanding the influence of anatomy on function such as measured by EEG or fMRI.

## Introduction

The relationship between structure and function has long been debated. Is a bigger brain more active? Is the shape of certain structures important? The advent of medical imaging, in particular magnetic resonance imaging (MRI) and even more diffusion (dMRI), has substantially transformed the way researchers address these questions. Structure and function cannot be separated from one another, and we can now investigate the relation between the way the brain is divided into substructures (segregation) and the way these areas communicate with one another (integration) [1].

Technical advances in the field of MRI and dMRI have enabled researchers to investigate how voxel-scale metrics—such as gray (GM) and white (WM) matter density, fractional anisotropy (FA), mean, radial or axial diffusivity and axonal density—are related to behavioral measures. In healthy subjects, for example, this has been addressed by comparing how such structural variations in the same age-group are related to memory, visual or motor skills [2–4]. However, few studies have analyzed the effect of more macro-structural information such as size, shape or position of WM and GM structures in healthy subjects (for a complete review, see Kanai and Rees [5]). Indeed, this topic is often addressed in healthy aging [6] or in patients with neurological disorders, where changes in shape may be more obvious, for example due to malformations. Here, we consider structural and functional variations across healthy subjects.

A particularly interesting case of functional variability is that derived from human electroencephalography (EEG) recordings. The alpha rhythm, the most prominent EEG rhythm, was first defined by Berger (1930) as EEG oscillations between 8 and 13 Hz, visible from the occipital cortex when the subject is awake with eyes closed and attenuated with eyes opening. In general, EEG alpha variability has been shown to be stronger across subjects (inter-subject variability) compared to repeated measures in the same subject (intra-subject variability) [7]. Yet, despite decades of research, questions regarding their *raison d’être* and origin are still debated. There have been multiple studies investigating alpha variability in healthy subjects with non-anatomical factors, such as cognitive behaviour, IQ, age and genetics (see Bazanova and Vernon [7] for a review) but, again, few have analyzed the effect of anatomical factors, such as the morphology of GM and WM components in the brain (size, shape, position, etc.).

Structures especially worth investigating are the thalamus, in particular the visual thalamus, and the associated white matter tracts, i.e. the optic tract (OT) and the optic radiation (OR), connecting the retina and visual cortex to the lateral geniculate nucleus (LGN) of the thalamus. Indeed, early microelectrode recordings in animal models showed that neural activity in the LGN is related to cortical alpha activity [8]. Later work in humans showed that spontaneous fluctuations in alpha power are correlated to thalamic blood oxygen level dependent (BOLD) functional MRI (fMRI) [9] and glucose metabolism derived Positron Emission Tomography (PET) (see Liu et al. [10] for a review). Also, knowing that the anatomy of the thalamus and its nuclei influence other phenomenon or diseases, many of which are related to vision (amblyopy, albinism, etc. [11, 12]), it is therefore plausible that such structural variations affect alpha waves, also vision-related. Andrews et al. [13] showed that, although the visual cortex, optic tract and LGN volumes are correlated together and vary between subjects by 100-200%, they are however independent from brain size, which varies by 18%. In terms of the LGN fiber pathways (OR and OT), it has been suggested that it could also influence alpha activity. On the one hand, increasing myelination increases the speed and efficacy of signal conduction, which could in turn promote neural synchrony at its cortical targets, thus influencing EEG measurements at the scalp [14]. On the other hand, variations in OR/OT volume might result in strong volume-conduction effects due to anisotropy [15], while fiber length and/or density could also affect scalp EEG measurements [16]. Yet, little work has analyzed the effect of thalamic structures on EEG alpha waves, which is surprising considering that most of the EEG rhythms generation models using the thalamus use such anatomical parameters [17], and considering that it has been proposed that effect of thalamic and OR variation should be considered [17, 18]. Nunez et al. [19] initially proposed that peak alpha frequency would be related to head size, though this result was recently challenged by Valdés-Hernándes et al. [17], who suggested that FA in sections of thalamic-related fibers was a better alpha correlate. However, given the well-known limitations of the tensor model (from which FA is measured) when quantifying WM, it is unclear whether such results hold when using reconstruction methods that are less sensitive to this limitation, such as High Angular Resolution Diffusion Imaging (HARDI) [20]. To our knowledge, only one study has analyzed the effect of thalamic GM/WM morphology on EEG: Hindriks et al. [21] used DTI connection strength and biophysical models to analyze the importance of the OR. However, connection strength uses the number of streamlines produced by tractography, which might not always accurately describe WM tracks [22]. Also, they could not analyze the effect of the LGN, a satisfactory segmentation of the LGN being difficult to obtain. We believe HARDI-based tractography probabilistic tracking handles the uncertainty in the principal directions extracted from the fiber orientation distribution functions (fODFs) and allows for bundle reconstruction with a higher spatial extent or volumetric coverage. Therefore, in the current study, we combine EEG, fMRI and HARDI-based dMRI to improve quantification of the OR and segmentation of the LGN and to investigate whether structural inter-subject variations in the LGN and its efferent fibers (OR) explain alpha variability in healthy humans.

### Background

#### Segmentation of the thalamus

The thalamus represents a mixture of white and gray matter, and thus has proven difficult to subdivide from structural images derived from T1 or T2-weighted MR sequences [36, 37], although specific techniques have been proposed [38–43]. Thalamic atlases exist, but, given the inter-subject variability of the thalamus and its nuclei [13], this may lead to inaccuracies. Diffusion MRI (dMRI) on the other hand may allow more precision in thalamic segmentation. The founding axiom of the thalamic nuclei segmentation algorithms based on dMRI is that different nuclei have different functions, and thus, different connections to specific parts of the brain. As a result, one could determine a nucleus by clustering the voxels belonging to an associated fiber [44, 45]. Indeed, several studies have used the diffusion tensor (DT) of each voxel to distinguish nuclei from one another without necessarily computing their fibers with tractography [44, 46–51], though this approach has drawbacks. There is the problem that DT precision is limited in areas with a high density of gray matter or in crossing fiber regions, such as the thalamus [24]. HARDI could help, but such algorithms have be shown to be less stable as they deal with more information [52]. Also, these clustering techniques make it difficult labelling nuclei automatically and often require visual expertise.

It then seems more promising to use tractography because we have more knowledge about the expected features of the fibers, such as their starting and ending points or their shape. The first articles proposing tractography for the segmentation of the thalamus used the structural connectivity to specific regions of the cortex [36, 45, 53–57], labeling the voxels with the name of the associated cortical region. However, first, instead of a thalamus segmentation problem, we then have to face a cortex segmentation one. Also, assessing the quality of the tractography is arduous, though some streamline probability scores have been proposed [32]. That problem will be even more important as HARDI probabilistic tracking becomes more popular, giving more precise streamlines, but along with more false positives [36, 58]. Also, these probabilistic metrics need an additional supervised thresholding step to ensure a valid segmentation. We therefore use the structural connectivity through one well known fiber, the OR, to segment the LGN.

#### Segmentation of the optic radiation

The OR has been studied intensively (see Table 1), mainly because of the high risk of vision loss after damages to the OR during temporal lobe epilepsy surgery or tumor resections. The OR originates from the LGN and leads to the occipital lobe after looping anteriorly from the LGN into the temporal lobe, around the temporal horn of the lateral ventricle (Meyer’s loop). It is generally believed that the bulk of the OR leads to the calcarine fissure (in the primary visual cortex, V1), though it has been shown recently that it may reach V2 and V3 as well [59].

**Table 1:** Litterature review, after 2005.

Year	Author	DTI Tracking?	Need LGN?	Other seeding ROIs	Inclusion ROIS	Exclusions ROIs
**2016**	**This study**	HARDI, P	Anterior thalamus	From FreeSurfer, modified	Visual cortex	AR, superior ROI, CLH, comparison to a template
**2015**	[23]	HARDI, P	✓	OC, V1	–	Fornix
**2015**	[24]	HARDI, P	Whole thalamus	From FreeSurfer, modified	Visual cortex	Low tracking density voxels, CSF, CLH, ipsilateral GM regions other than seed and target masks
**2014**	[25]	✓, D	✓	M, from RGB	–	ROI at the pons and CST
**2014**	[26]	✓, SP	✓	(6 different segmentations)	SS (3 different segmentations)	–
**2014**	[27], 1	HARDI, D	–	TL and OL	OL and LGN (posterior thalamus)	AR, CST and other ROIs if needed
**2014**	[27], 2	HARDI, P	–	Whole brain	LGN from a first rapid OR tracking and OL	AR and fibers with low connectivity score
**2011**	[28], 1	MT, P	WM near LGN	M, from FA+PD	–	AR and fibers with low connectivity score
**2011**	[28], 2	MT, P	✓	From OT	V1, M	–
**2011**	[29], 1	✓, D	✓		V1	AR
**2011**	[29], 2	✓, D	✓	RELIRE?	–	–
**2009**	[30]	✓, D	✓	M, from FA	V1 and OL	–
**2009**	[31]	MT, P	WM near LGN	M, from FA+PD	–	AR
**2008**	[32]	✓, P	✓	From OT	V1	Fibers with low connectivity score
**2007**	[33]	✓, P	–	Whole Brain	OL and LGN, M	–
**2007**	[34]	✓, P	–	Whole Brain	OL and SS, M	2 ROIs beside Meyer’s loop, M
**2006**	[35]	✓, D	✓	M, from b0	OL, Green on RGB	–

PD = principal direction of the tensor, RGB = colored FA

DTI = diffusion tensor imaging, MT = multi-tensor, HARDI = high angular resolution diffusion imaging

P = probabilistic, D = deterministic, SP = streamline probabilistic

OT = optic tracks, OC = optic chiasm, OL = occipital lobe TL = temporal lobe, SS = sagittal stratum, CST = cortico-spinal tract, AR = anterior ROI, CLH = contra-lateral hemisphere

M = manually

OR tractography often depends on a roughly defined LGN from a priori information, a region of interest based on the fractional anisotropy (FA) or on the principal diffusion direction (PDD) of the DT, or manually segmented (see Table 1). However, for this study, we have two objectives: the analysis of the OR and the analysis of the LGN. We are looking for the opposite of what has been published: we want to obtain the LGN from a good segmentation of the OR using tractography. We develop a nearly-automated fiber analysis tool that we believe gives reliable results, based on fiber comparison to a template.

With new information on the size and shape of the thalamus, the optic radiation and the associated nuclei, the LGN, we address the question of the influence of structure on EEG alpha waves. We show that our semi-automatic method using probabilistic tracking allows obtaining good optic radiations. We also show that in this case, structure alone is not sufficient to explain variability in the alpha power or frequency.

## Methods

### Subjects

Twenty-three healthy subjects were recruited for the study (12 males). All subjects were native French speakers with no psychiatric or neurological symptoms. The study was approved according to the guidelines of the Internal Review Board of the Centre Hospitalier Universitaire de Sherbrooke (CHUS) and all participants provided written consent. Data from two subjects was discarded due to excessive movement during MR acquisition. Another subject showed no EEG alpha peak, and was also excluded.

### Data acquisition

MR Imaging data were acquired using a 1.5 T SIEMENS Magnetom (Vision). Noise-reduction headphones and head cushions were used to minimize artifacts. Each session started with an anatomical T1-weighted 1mm isotropic MPRAGE (TR/TE 1860/3.54 msec) acquisition, followed by a fMRI protocol and finally, with a dMRI acquisition. Functional MRI datasets were collected using a standard echo-planar imaging (EPI) sequence: 35 axial image slices, 64 x 64 matrix, TR/TE 2730/40 msec, voxel size 3.438 x 3.438 x 4.2mm. Data were acquired in a box-car format, with subjects alternating between baseline and task conditions via short auditory cues. Subjects performed alternating epochs of 30 sec rest, eyes closed, and 20 sec eyes open, repeated 5 times and ending with a rest epoch, resulting in a total scan time of 4 min and 40 sec. Diffusion MRI datasets were acquired using a single-shot echo-planar (EPI) spin echo sequence of 12 minutes (TR/TE = 11700/98 ms), with b-value of 1000 s/mm^2^, 64 uniform directions, matrix size of 128x128, 2mm isotropic spatial resolution. To reduce susceptibility distortions, GRAPPA parallel imaging was employed with an acceleration factor of 2.

EEG data were acquired on the same day, for each subject, using a 64-channel EEG system (Brain Products) with sampling rate of 500Hz, according to the 10-20 system, in an eyes closed-eyes open (EC-EO) task guided by an auditory cue (30 seconds EC, 20 seconds EO, for a total recording time of 5 minutes).

### Data preprocessing

**Fig 1:**
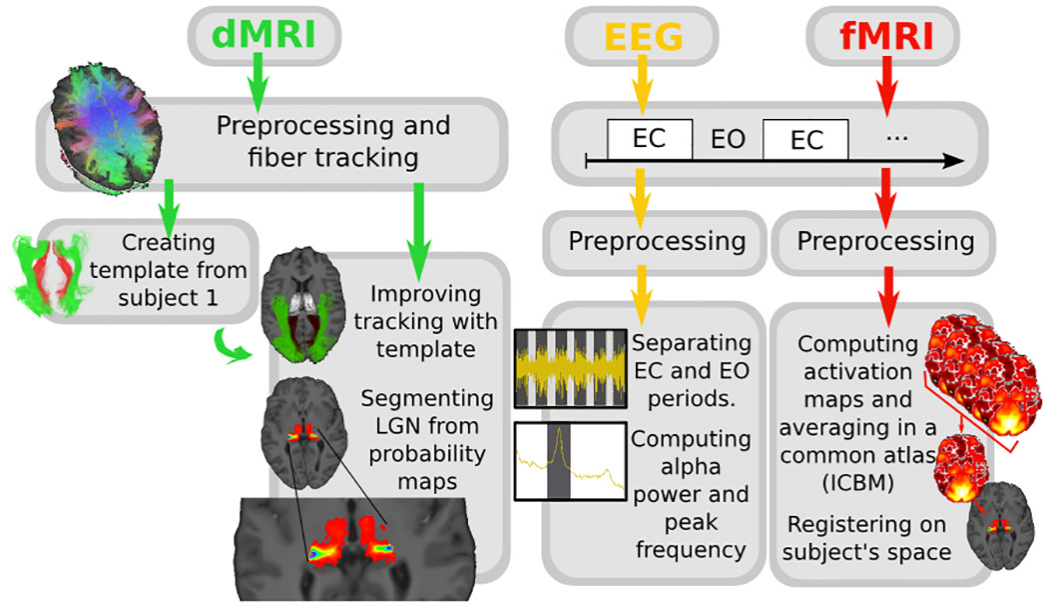
Analysis pipeline describing dMRI, fMRI and EEG analysis.

All preprocessing and processing steps are reflected in Fig 1.

#### T1-weighted images

T1 images were denoised using non-local means (NLM) [60] (Dipy, [61]) and skull-stripped using FSL [62]. T1-weighted images were registered to the upsampled b0 (1x1x1 mm); linear registrations were first performed with FSL FLIRT [63], followed by non-linear registration with ANTS [64]. Non-linear registration was useful even though it is an intra-subject registration to compensate for dMRI deformations.

#### Diffusion-weighted images

Diffusion images were denoised using NLM with Rician distribution adaptation [65] available in Dipy. Datasets were upsampled to 1mm isotropic resolution with trilinear interpolation. The b0 image was extracted using AFNI [66] and skulled-stripped using FSL [67]. Tensors, fractional anisotropy (FA) map and fODFs were computed using Dipy.

#### Functional images

The description of all fMRI analysis can be found in [68]: We processed the data using slice timing and motion correction, band-pass temporal filtering (0.008 to 0.1 hz) and detrending. The spatial smoothing was replaced by NLM denoising (Dipy). The activation maps were generated by computing the Pearson correlation coefficient between the hemodynamic response function (HRF) convolved stimulus box-car and the voxel time-series. The activation were then converted to z-scores and thresholded at the 96th percentile (equivalent to a z-score of ∼2.5). We then registered the activation maps to the T1-weighted image using the same procedure as described previously (FLIRT+ANTS).

#### EEG data

EEG preprocessing was performed using EEGLAB [69]. EEG signals were high-passed filtered at 0.5 Hz. ICA was computed, and bad components were identified as follows. First, the z-score of the power spectral density (PSD) between 5 and 18 Hz was computed. If no point in the alpha band (8-13 Hz) had a z-score higher than 1 (i.e. the component has no alpha peak), the component was removed. Second, the z-score of the weights of the component on each electrode in the mixing matrix was computed. If a component had a z-score higher than 4, it was removed. Thresholds 1 and 4 were chosen after visual analysis of the data. Further details can be found in [70]. For three subjects, one or two channels were interpolated (due to excessive noise) prior to ICA decomposition. Data was rereferenced to an average reference.

### Data processing

#### Brain structures

The size of each brain was measured as proposed by FSL-FAST (http://fsl.fmrib.ox.ac.uk/fsl/fslwiki/FAST). Thalamic masks and occipital lobe masks were obtained using FSL FIRST [71] and FreeSurfer (http://freesurfer.net/), respectively. Thalamic masks were further modified by constraining them to voxels whose FA was in the range [0.1, 0.5] in order to remove parts of the cortico-spinal tract (CST) that were incorrectly included in the original segmentation, as proposed by Mang et al. [56].

#### Tracking

Tracking was performed separately for the left and right thalami. Seedpoints were placed in the posterior voxels of the thalamus, found automatically as the 3/4 posterior portion between the most anterior point and the most posterior point. Both deterministic and probabilistic tracking were performed with streamtrack from MRtrix [72], with default values, on a FA > 0.1 mask, giving 50*n* unidirectional tracks where *n* is the number of voxels in the seeding region, excluding tracks crossing the anterior part of the thalamus. In most of the articles working on the segmentation of the OR, DT deterministic is used (see Table 1). However, the use of HARDI probabilistic tracking has proven useful, allowing reconstructing a larger extent of the OR, namely a longer Meyer’s loop [27]. Tracking results were then filtered, keeping only streamlines touching the whole occipital lobe mask. Streamlines reaching the opposite hemisphere or the anterior part of the brain were discarded with exclusion boxes.

#### OR and splenium templates

**Fig 2:**
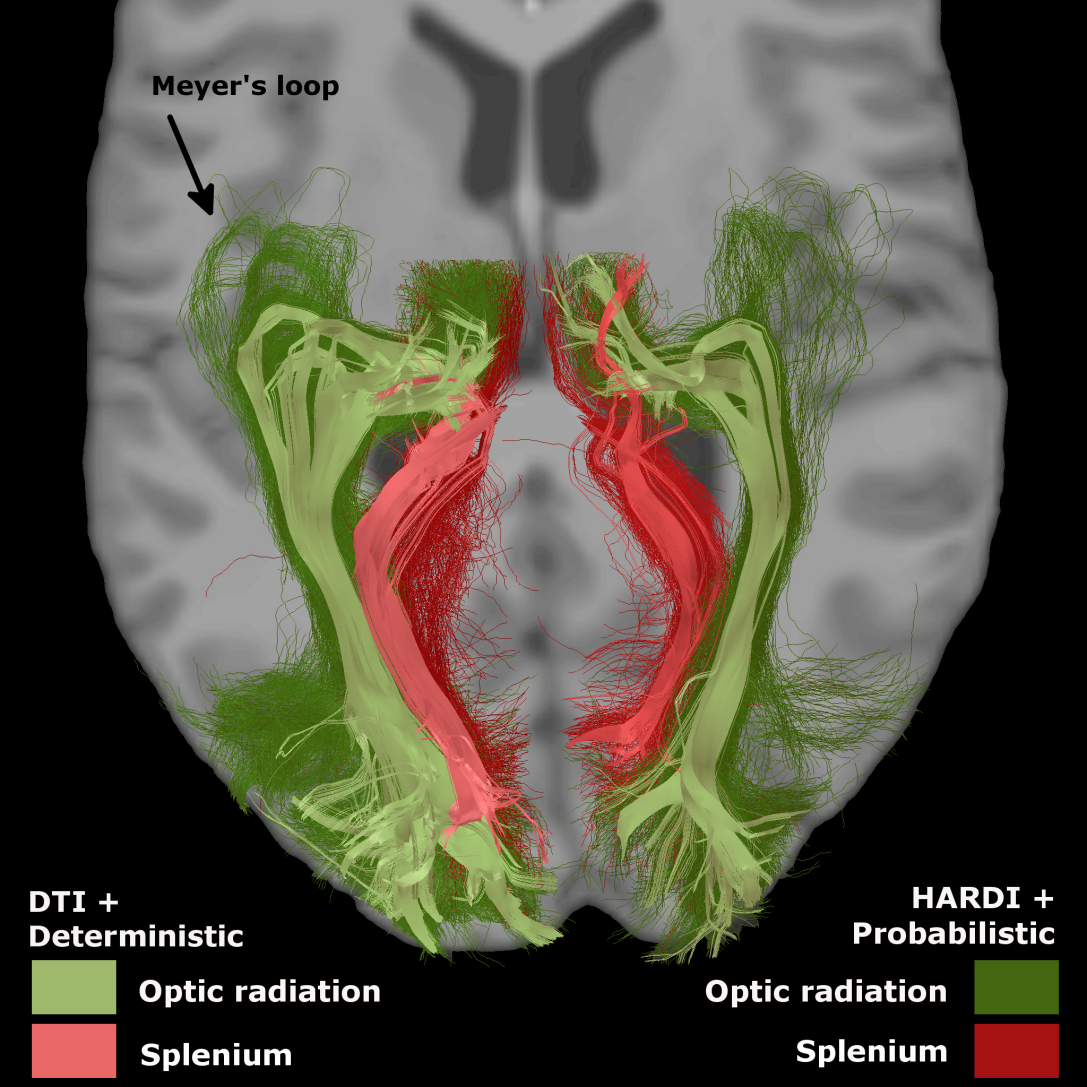
Templates of OR (green) and splenium (red). The difference between deterministic + DTI tracking (paler) and HARDI + probabilistic (darker) was major, mainly in the Meyer’s loop. Probabilistic template was used.

**Fig 3:**
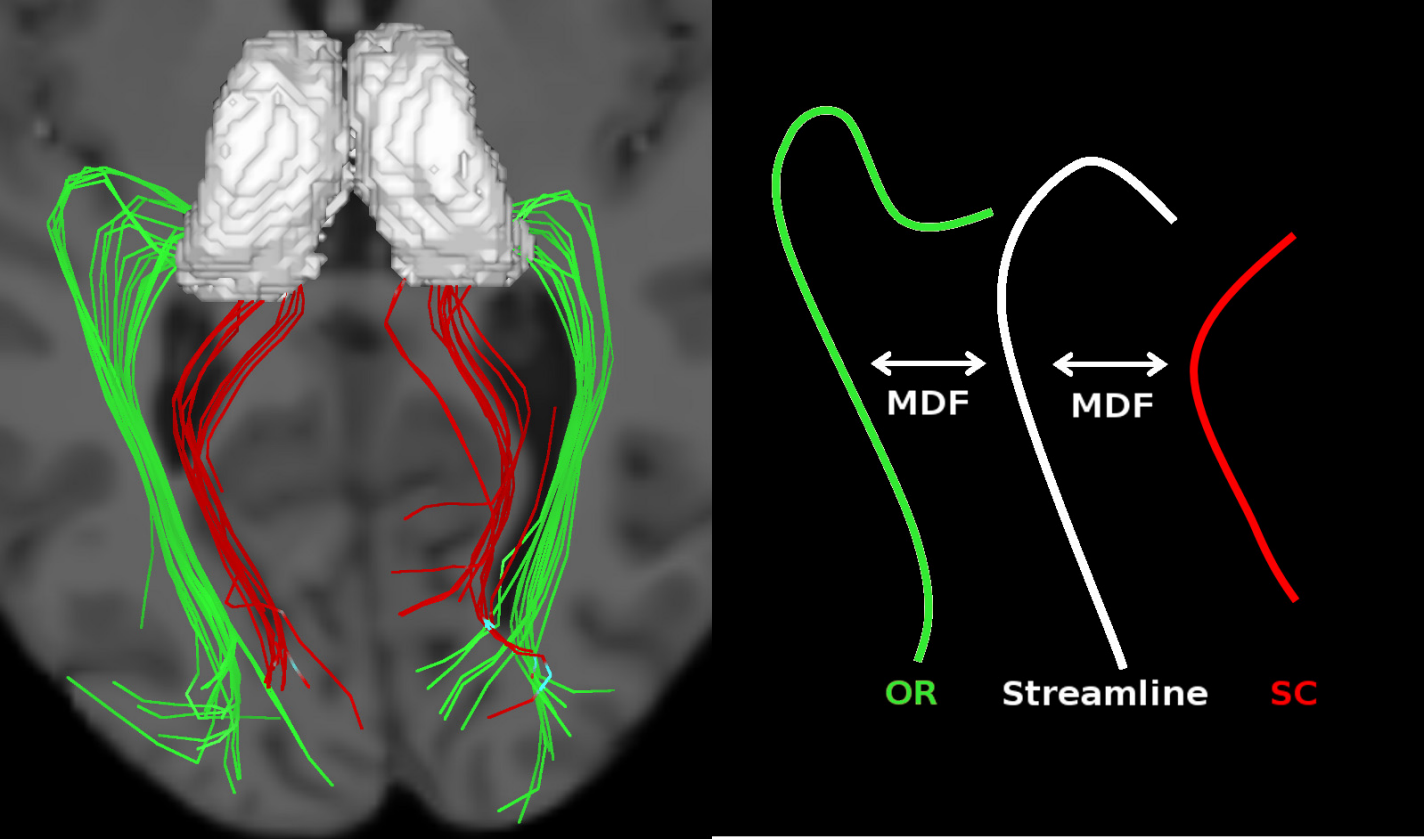
Templates (on the left) and fiber comparison with MDF (on the right). Each streamline of the subject (white) is kept if it resembles the OR template (green), rejected otherwise (cases were it resembles the splenium template (red)).

We observed that the tracking results included part of the splenium of the corpus callosum (SCC). To correct for this, we manually created templates for each bundle—the OR and the SCC—by visual inspection of subject 1’s tractography result. Subject 1 was chosen randomly because his tractogram was standard, i.e average quality not no apparent erroneous results. Here, we kept tracks that broadly resembled either the OR and SCC (see Fig 2, on the left) such that they could be quickly identified in the remaining subjects. The bundles were subsampled using QuickBundles, a clustering algorithm from Dipy, to keep only a few representative streamlines, acting as the templates, as indicated in Fig 3.

#### OR segmentation and metrics

The OR bundle of each subject was automatically segmented by comparing each streamline of the subject’s tracking result to the OR and SCC templates. Templates were first registered to the diffusion space of that subject by a linear registration. The registration matrix was defined by FLIRT, using a mask combining the two thalami and the occipital lobe. We chose this mask because it was only important to have the posterior part of the brain aligned with the subject’s space, since it contains the OR. The comparison between the streamlines and the templates was performed with the Mean Direct-Flipped (MDF) metric from Dipy. If MDF(streamline, OR) was smaller than MDF(streamline, SCC), the track was kept. Otherwise, it was removed (see Fig 3).

**Fig 4:**
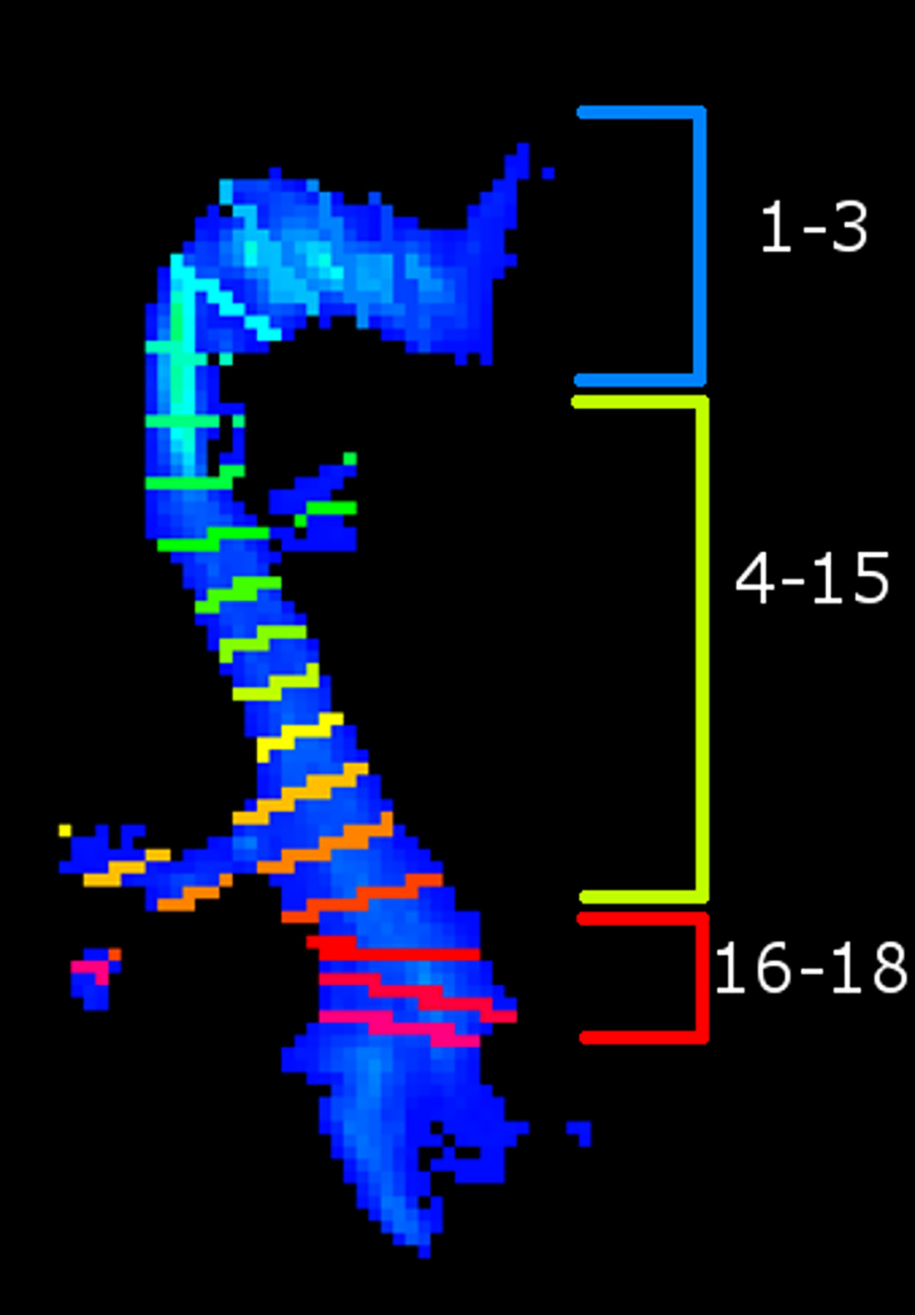
Superior view of the OR. Cross section areas (CSAs) were defined as the number of voxels where passed at least 5 streamlines in each section. Mean and minimum CSA of sections 4-15, and mean and maximum CSA of sections 16-18 were measured.

Mean FA and apparent fiber density (AFD) were measured [73, 74], as well as distances between the anterior tip of the temporal horn and the anterior tip of Meyer’s loop in ICBM space (TH-ML distances). TH was selected visually (y = 86) and ML was defined as the most anterior (y) point with at least 5 streamlines. This criteria was added to avoid remaining spurious tracks. Streamline count was computed at each step of the process (initial tracking, after filtering the streamlines reaching the occipital lobe only, after deletion of the SCC). However, to characterize the final OR, streamline count was not used used to quantify the OR since this measure is known to be easily biased [75, 76]. Rather, we computed the mean cross-sectional area (CSA), which is a more robust tract-based metric as it takes into consideration the shape (size, length) of the fiber bundle. This technique is already used in histology [13] and is becoming popular with tractography [77]. We selected 18 equally spaced plans perpendicular to the bundle (sections). We discarded the three first ones since they were too close to the thalamus (see Fig 4). For each section, the CSA was defined as the number of voxels in that plane where passed at least 5 streamlines. The mean CSA was computed for sections 4-15, as well as the minimum CSA, acting as a measure of the stem of the OR. Statistics on the three last sections (16-18), where the OR bundle significantly spreads out, were computed separately to measure the spanning of the OR in the occipital lobe: the maximum and the mean CSA were computed. If a voxel was included in more than one plane, it was counted only once, in the plane closest to the occipital cortex. Voxel count was prefered to other measures such as radius to prevent artificially increased CSAs in spanning areas (ex. planes 13 and 14 in Fig 4).

#### LGN segmentation

For each subject, the number of OR streamlines in each voxel was transformed into a density map with MRtrix. Voxels outside the thalamus in the OR density map of each subject were set to zero, and the z-scores of the non-zero voxels were calculated. We defined the LGN of each hemisphere as the voxels with z-score higher than 4. This threshold was chosen to obtain LGN sizes corresponding to the sizes presented in the literature (115mm^3^ on the left and 131mm^3^ on the right [13]). The sizes with threshold 4 or with other thresholds were highly correlated, and thus, the threshold value does not influence the final correlations with EEG alpha metrics. LGNs were also registered on ICBM452 standard space [78]. An occurrence map (in percentage of subjects) was obtained in the ICBM space with, for each voxel’s value, the number of subjects whose LGN touched this voxel.

#### EEG metrics

EO and EC windows were separated, and the PSD was computed for each window separately. PSD estimates were then averaged separately for the EO and EC conditions. Data were averaged over all P, PO and O electrodes. EC maximum and average power in the alpha band (8-13 Hz) over these electrodes was measured, and same in the spectrum obtained by subtracting the EO spectrum from the EC spectrum, such as was done by Posthuma et al. [79]. The peak frequency, or alpha peak (P_*α*_), was defined as the frequency of the maximum EC power. The same results were measured for the left occipital electrodes and for the right occipital electrodes separately.

#### fMRI metrics

The activation map of the eyes open-closed task was obtained by computing the mean across each subject’s activation map and thresholding it for z-scores 2.5 and higher.

## Results

### Thalami masks

**Fig 5:**
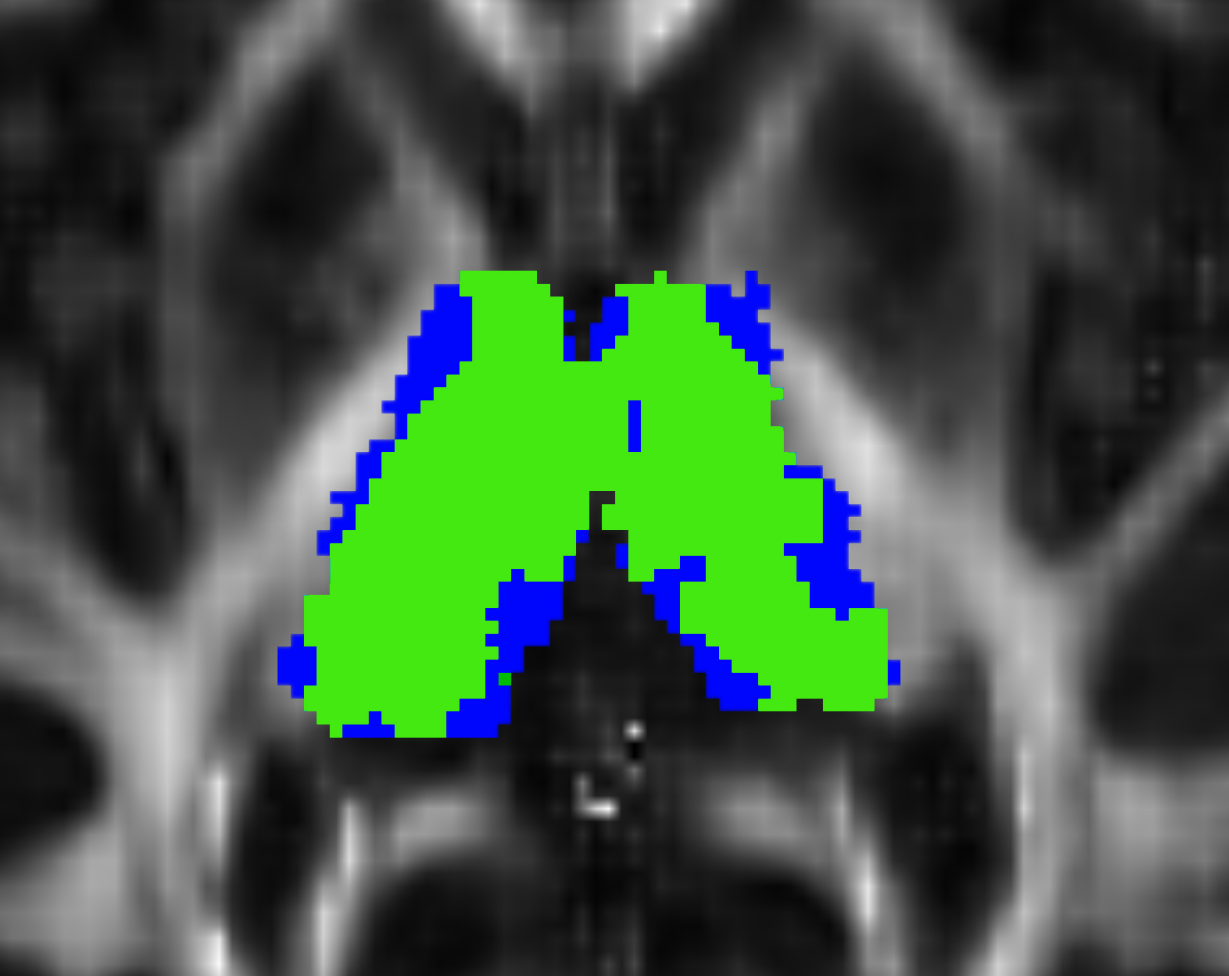
Thalamus mask modification. Blue: thalamic masks as defined by FSL. Green: voxels where FA ∈[0.1, 0.5]. Background: the FA map.

The thalami mask had a mean size of 9450 mm^3^ ± 1158 in the left hemisphere and 9110 mm^3^ ± 1044 in the right, representing 81.4% and 85.1% of the mask given by FSL, respectively (see Fig 5).

### Tractography, templates and OR segmentation

**Fig 6:**
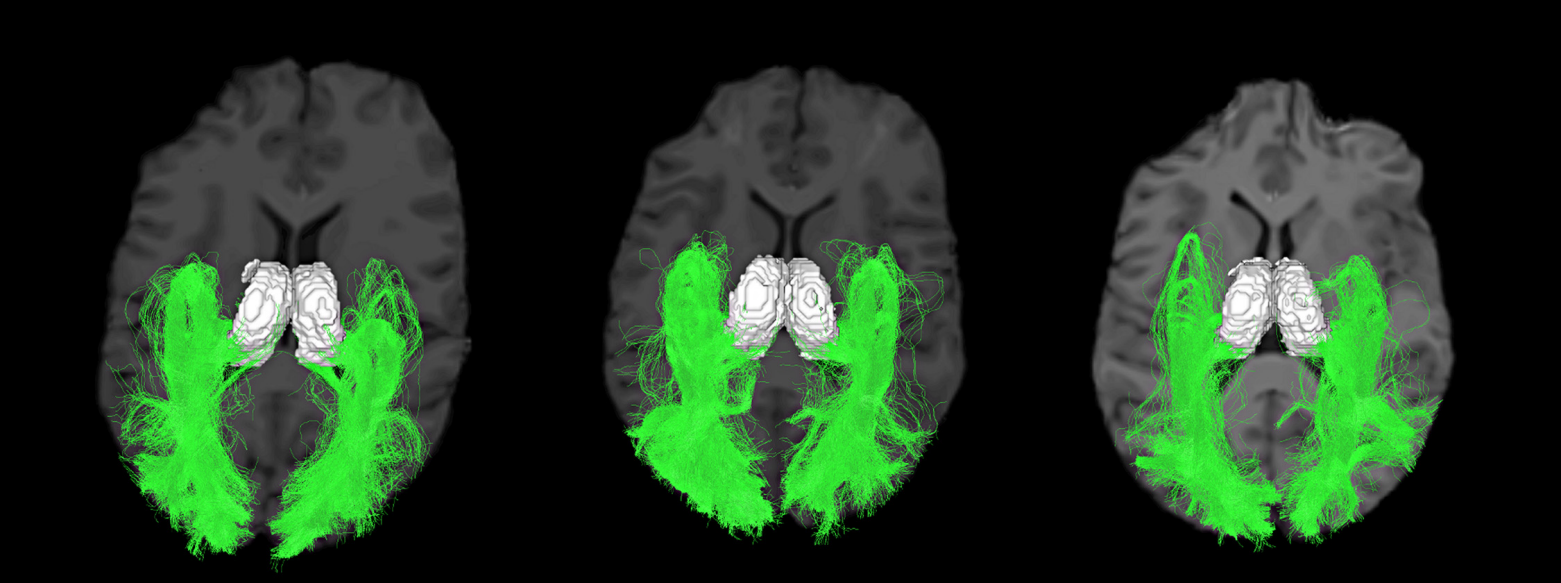
Three subject’s final OR, axial view.

Tracking results showed a large Meyer’s loop and a wide spanning of streamlines in the occipital lobe (see Figs 2 and 6). Number of streamlines touching the occipital area represented, in the left and right hemisphere, 3.3% ± 0.9 and 2.4% ± 0.7 of the initial tracking. The final number of streamlines in the OR (SCC deleted) then represented 70% ± 13 and 48% ± 17 of the occipital-touching ones. Results were similar with deterministic tracking. Cross-sectional areas (CSAs) are reported in Table 2 and represented in Fig 4. Although the fiber count indicated a much bigger left OR than right (2.7 times bigger, p < 0.1e^−6^), the mean CSA was more similar (left was approximately 1.3 times larger than right, p = 0.02). Also, left and right OR CSAs were correlated (p < 0.0005), while fiber count was not (p = 0.7). The average FA and AFD within the OR are shown in Table 3. TH-ML distances were of −6 ± 3 mm on the left and −4 ± 3 mm on the right (negative results are anterior to the tip of the temporal horn). TH-ML distance with DTI and deterministic tracking were, respectively, of −2 ± 4 mm and 2 ± 5 mm.

**Table 2:** Cross-sectional areas (in number of voxels).

	Left hemisphere		Right hemisphere	
Planes	4-14	15-18	4-14	5-18
**Min**	179 ± 39	–	130 ± 35	–
**Min**	247 ± 56	357 ± 106	191 ± 55	207 ± 68

**Table 3:** FA and AFD under the OR.

	Left hemisphere	Right hemisphere
**FA**	0.38 ± 0,02	0.39 ± 0,03
**AFD**	0.77 ± 0.03	0.77 ± 0.04

### LGN segmentation

**Fig 7:**
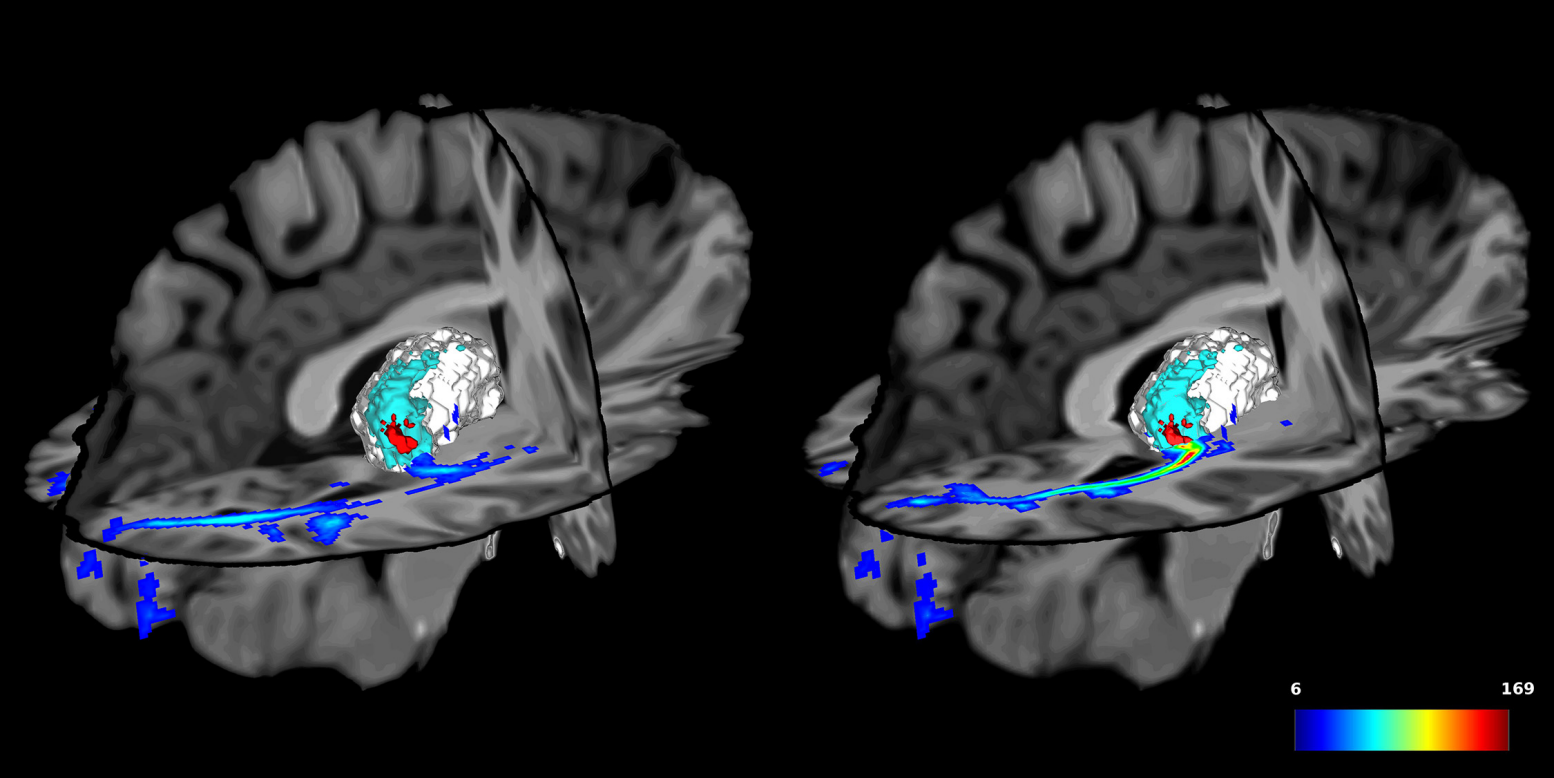
Effect of the thresholding on the ORs. In the thalamus: in blue, the final OR, voxels crossed by at least 5 fibers and in red, with a threshold on the z-score of 4. In the optic radiation: density map, in number of streamlines. On the left: z = 64. On the right: z = 69.

LGN sizes obtained from the thresholded density map (see Fig 7) were of 116 ± 18mm^3^ on the left, and 100 ± 26mm^3^ on the right. The position of the centroid of the LGN (the point with the strongest density, i.e. the most streamlines), varied very little across subjects, as shown in Table 4.

**Table 4:** Position of the strongest density point, in ICBM space.

	Left hemisphere	Right hemisphere
**x**	95 ± 3	56 ± 1
**y**	86 ± 1	84 ± 2
**z**	73 ± 5	68 ± 4

### EEG alpha waves measurements

Visual inspection confirmed that no subject presented a peak outside the 8-13 Hz range. All of these measures were also similar across hemispheres, as shown in Table 5 (p >0.05 in all cases).

**Table 5:** EEG results.

	Left hemisphere	Right hemisphere	Posterior
**Alpha peak frequency (Hz)**	10.4 ± 0.8	10.6 ± 0.9	10.5 ± 0.8
**Mean EC power** (*μV*/*Hz*)	16.8 ± 6.0	18.4 ± 6.2	18.7 ± 5.9
**Mean alpha power (EC-EO)** (*μV*/*Hz*)	16.3 ± 5.2	16.8 ± 4.8	17.5 ± 4.7

### Relation with fMRI results

**Fig 8:**
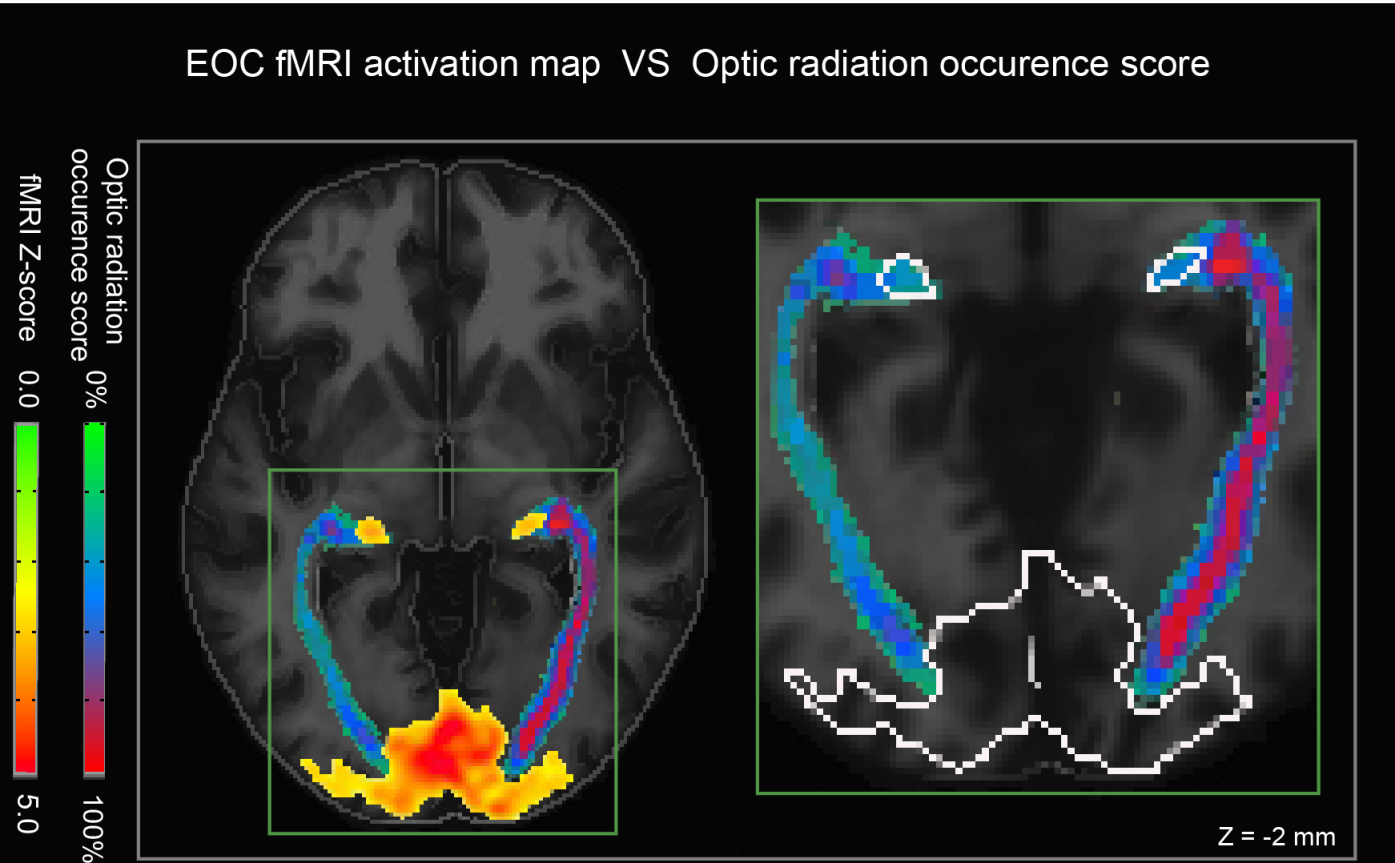
Validation of LGN segmentation. (left) The red/yellow overlay represents fMRI activity during the EC-EO task. Note the widespread activation in the visual cortex and more focal bilateral activation in the thalamus (LGN). (right) An enhanced view shows that our reconstructed streamlines converge nicely onto the fMRI activation sites (outline in white).

To assess the quality of our LGN position, we visually compared the occurence maps of the OR in the ICBM space with the maps of the mean fMRI activation of all subjects. Thalamic voxels with highest occurence score were localized near the fMRI activation sites, as shown in Fig 8.

### Link between structure and EEG alpha waves

Overall, we computed 68 Pearson correlations and associated p-values between EEG alpha and dMRI measures in each hemisphere (see Table 6). All scatter plots were visually inspected to ensure that these results were not driven by outliers, and the smallest p-value was 0.06.

**Table 6:** p-values for all combinations between EEG data and structural data. Smallest value was 0.06.

	Max power (EC-EO)		Alpha peak		Max power (EC)	
	L	R	L	R	L	L
**Thalamus**						
Size	0.76	0.43	0.66	0.34	0.91	1.00
Size (as % of the brain)	0.51	0.34	0.45	0.27	0.93	0.67
Size (left minus right)	0.63		0.19		0.75	
**Streamlines**						
Number	0.49	0.23	0.74	**0.06**	0.77	0.40
Number (left minus right)	0.35		0.11		0.51	
Most anterior y point	0.29	0.42	0.45	0.15	0.15	0.65
Average length	0.35	0.50	0.92	0.21	0.66	0.77
**Cross-sections**						
Average area	0.38	0.30	0.16	0.27	0.90	0.26
Average area (as % of the brain)	0.36	0.36	0.18	0.29	0.84	0.30
Average (left minus right)	0.94		0.86		0.32	
Min	0.68	0.43	0.13	0.26	0.80	0.32
Max	0.45	0.95	0.53	0.09	0.42	0.68
**FA and AFD**						
FA	0.96	0.64	0.63	0.98	0.88	0.93
AFD	0.60	0.47	0.52	0.28	0.46	0.11
**LGN**						
Initial size	0.96	0.16	0.80	0.35	0.61	0.12
Initial size (as % of the thalamus size)	0.71	0.19	0.68	0.25	0.98	0.14
Size (with threshold on z-score)	0.73	0.98	0.49	0.34	0.86	0.27
Idem (as % of the thalamus size)	0.91	0.97	0.40	0.35	0.55	0.43
Idem (left minus right)	0.50		0.45		0.35	
**Meyer’s loop**						
Distance tip of the temporal horn to tip of Meyer’s loop	0.29	0.42	0.45	0.15	0.15	0.65

## Discussion

The main goal of this study was to investigate the relationship between structure and function in the brain by considering the influence of thalamus and optic radiation (OR) morphology on EEG alpha waves. To do so, we developed a novel approach for quantifying subject-to-subject variations in different measures of thalamic and OR structures with a nearly-automated fiber analysis tool. The selection of the ROIs to perform the tractography is automatic. The sorting of the OR streamlines uses templates that can be defined by a non-expert. The comparison of the streamlines to the templates is automatic and does not need thresholds. Our method allowed us to obtain precise OR to isolate the LGN in multiple subjects, proving its efficiency. Nevertheless, no significant correlation with EEG was observed.

### Structure

#### Optic radiation

The OR is a well-studied fiber bundle. In humans, it is known to emerge from the LGN, to display a relatively large loop that partially extends into the anterior temporal lobe (Meyer’s loop), and to end as the inferior sagittal stratum in the visual cortex. Although it is made of one single sheet, it is constituted of three parts, each of them having a different curvature [26].

Our reconstructed tracts also follow this pattern (Fig 6). To ensure recovery of the whole OR, we used few anatomical priors, placing a large ROI for the seeding points (3/4 of the thalamus, with no condition on the initial direction), and using the whole occipital cortex as target region for the streamlines. Probabilistic tracking allowed the streamlines to successfully follow the OR, even in the highly curved sections, without additional ROI. The fact that the occipital-touching streamlines represented only around 2-3% of the initial streamlines highlights the difficulty in recovering the OR with tractography. This number would be smaller with whole brain tractography, and an extremely large number of initial seeds would be needed before obtaining a satisfactory OR. For this reason, tracking from the thalamus allows better results, though it may include half of the splenium if the initial thalamus segmentation is overly conservative. Our fiber comparison to a template allowed differentiating the OR bundle from the SCC.

Distances between the anterior tip of Meyer’s loop and the anterior tip of the temporal horn were comparable to other literature reports. Indeed, in Ebeling and Reulen [80], TH-ML distances in cadavers were of -5 mm in average, which is similar to our results. Few *in vivo* studies have reproduced this [27, 81]. It might be due to the use of probabilistic tracking, as discussed by Lilja et al. [27], but also to the use of HARDI instead of DTI. Indeed, these distances with our DTI and deterministic tracking were smaller (i.e. the Meyer’s loops were less anterior). Also, as reported by others [23, 25, 27], left Meyer’s loop (ML) was more anterior, as revealed with the TH-ML measure, and left OR was bigger, in the streamlines count, but also, to a smaller degree, in the average cross-sectional area (CSA). Our methodology permits different measures, in addition to the cross-sections (e.g. circumference), though this is outside the main scope of our study and remains an active field of research for bundle-based and tract-based metrics. In summary, our OR reconstruction exhibits properties that closely resemble its known anatomy despite the fact that the diffusion images were acquired at relatively low field strength (1.5T). Increasing the spatial resolution is unlikely to affect our results as we purposely focused on more macroscopic measures of tractography.

#### Density maps and LGN

Our track density maps appeared to peak near the LGN, which was then confirmed using fMRI (Fig 8). The peak density location was very stable across subjects (see Fig 6), suggesting that our approach can not only accurately localize the LGN, but also that these 3D coordinates may be included as an anatomical reference in a standardized space, such as the ICBM.

However, it should be noted that the nearby pulvinar, whose main projections mainly target visual areas V3 to V5, also show connections the V1 and V2 [82]. It is therefore possible that some of our reconstructed fibers may also arise from the pulvinar and it is perhaps more accurate to label our purported LGN area as “visual thalamus”. We are currently working toward a more accurate segmentation of the LGN and pulvinar.

We should also note that LGN size depends most probably on the initial thalamus size, in itself a research subject [37, 83].

### Function

For all subjects, the peak alpha frequency was, as expected [7], situated between 8 and 13 Hz, and all subjects showed a robust change in power during EO-EC in occipital/parietal electrodes, revealing a good control of the signal to noise ratio. For our analysis, we used the maximum alpha power. For the average power, it has been suggested that a subject-driven band instead of the usual 8-13 Hz is more appropriate [7]. However, the use of average power was unnecessary here because it was highly correlated to the maximum and brought no additional information on the EEG variability across subjects.

### Structure vs function

No significant correlation was found, for any pair of metrics between structural metrics and EEG metrics (all p-values > 0.05), suggesting that OR and LGN structures have little influence on EEG alpha inter-individual variability. This idea is further supported by the fact that OR segmentation results showed lateralization, but not EEG. The reason behind this dissociation is unclear. On the one hand, our results are in contrast to those reported by Hernandez, who showed a significant correlation between tensor-derived FA of the posterior and superior corona radiata, probably associated to interactions between thalamus and cortex and in the posterior commissural fibers of the Corpus Callosum (splenium), and alpha peak frequency [17]. However, as stated in the introduction, such FA metrics are strongly biased by fibre crossings, which might explain our lack of correlation when using HARDI-based reconstructions. Other studies using voxel based morphology (VBM) have also reported low correlations (r = 0.1—0.3) between WM volume and alpha power [6, 84, 85], which is more in line with our findings, though it should be noted that VBM- and dMRI-derived measures of WM are based on very different phenomena. More work in how these two modalities are related could help in reconciling these discrepancies. For instance, it possible that certain microstructural properties of the LGN/OR/OT, such as myelin content [86], are better correlated to EEG than the WM metrics identified in the current study.

In summary, we here report on a novel approach for identifying the visual thalamus as well as its efferent fibre tracts. This method could be easily extended for accurately segmenting other thalamic nuclei and fiber bundles in different cortical areas, using templates in bundle atlases, for improving our understanding of structure-function relationships in humans.
